# SPP1 regulates tumor progression through modulation of signaling pathways and the tumor microenvironment

**DOI:** 10.1007/s12672-025-04273-6

**Published:** 2025-12-14

**Authors:** Yang Feng, Xuanbo Shao, Zhuo-Fan Xu, Penghao Liu, Huantong Wu, Yuanchen Cheng, Maoyang Qi, Hongfeng Meng, Boyan Zhang, Feng-zeng Jian, Zan Chen, Wanru Duan

**Affiliations:** 1 Neurosurgery Department, Capital Medical University Xuanwu Hospital, Beijing, China; 2 Capital Medical University, Beijing, China

**Keywords:** SPP1, Osteopontin, Tumor microenvironment, Tumor-associated cells, Tumor progression

## Abstract

Secreted Phosphoprotein 1 (SPP1), also known as Osteopontin (OPN), is a phosphorylated glycoprotein that plays a crucial role in regulating various cellular functions and immune responses. Recent studies have highlighted SPP1’s involvement in key physiological processes, including cell migration, proliferation, differentiation, and its significant impact on immune regulation and inflammatory responses. In the context of cancer, SPP1 is closely associated with tumor progression and prognosis. It contributes to tumorigenesis by promoting the formation of the tumor microenvironment (TME), influencing the behavior of tumor-associated cells, and enhancing tumor cell invasiveness and immune evasion. This review aims to provide a comprehensive analysis of SPP1’s role in the development and progression of various cancers, highlighting its potential as a therapeutic target and biomarker for cancer management.

## Introduction: discovery and biological functions of SPP1

### Identification and molecular characteristics of SPP1

SPP1, also known as Osteopontin (OPN), was first identified by Oldberg et al., who isolated a cDNA clone from rat osteosarcoma phages [1]. Their analysis revealed a 1473 bp sequence encoding a 317-amino-acid protein, which was named Osteopontin due to its binding properties with bone-specific sialoprotein. This discovery laid the foundation for understanding the molecular structure and biological functions of SPP1.

The SPP1 gene is located on chromosome 4q22.1 in humans, spanning 7.7 kb and consisting of 7 exons. The encoded human Osteopontin (hOPN) protein comprises approximately 314 amino acid residues and contains an N-terminal RGD (Arginine-Glycine-Aspartic Acid) sequence, which is crucial for cell adhesion by facilitating interactions with cell surface integrins [2–4]. Post-translational modifications, such as phosphorylation, play a pivotal role in modulating SPP1’s biological functions by influencing its binding affinity to receptors. Additionally, enzymatic cleavage of the SPP1 gene product generates fragments with diverse biological activities, allowing it to participate in multiple physiological and pathological processes [5, 6]. Both full-length SPP1 and its proteolytically cleaved fragments play complementary yet distinct roles in tumor progression: cleaved SPP1 fragments, by exposing cryptic integrin-binding motifs, exhibit enhanced pro-invasive and immunosuppressive activities through promoting cell adhesion, migration, and remodeling of the tumor microenvironment, whereas the full-length protein and its splice variants sustain tumor cell survival and stemness via CD44/integrin-mediated signaling. Therefore, optimal therapeutic strategies should aim to simultaneously block key receptor interactions and prevent the generation of oncogenic SPP1 fragments [7–9].

SPP1 is widely expressed in various tissues, including the gastrointestinal tract, inner ear, mammary gland, lung, salivary gland, kidney, and placenta. Functionally, SPP1 is a highly phosphorylated glycoprotein that contributes to numerous physiological and pathological processes, including cardiovascular diseases, cancer, diabetes, kidney stone disease, inflammation, biomineralization, cell viability, and wound healing. Notably, SPP1 promotes wound healing and cellular survival through multiple mechanisms, such as the regulation of growth factor and cytokine secretion, highlighting its critical role in tissue repair and homeostasis [4, 10, 11].

### Multifunctional roles of SPP1 in cell biology and immunity

SPP1 serves as a key regulator of cell adhesion, migration, and extracellular matrix (ECM) remodeling. By interacting with cell surface receptors, such as integrin αvβ3 and CD44, SPP1 facilitates cell adhesion to specific matrices, thereby playing a crucial role in ECM composition, remodeling, and tissue regeneration [3, 12, 13]. This interaction is particularly important for wound healing and bone remodeling, as SPP1 regulates the activities of osteoblasts and osteoclasts, thereby promoting bone mineralization and homeostasis [11].

Beyond its structural and regenerative functions, SPP1 is a multifunctional cytokine that actively participates in immune regulation by modulating various immune cells:

1. Macrophage Polarization: SPP1 promotes M2-type macrophage polarization, which is associated with anti-inflammatory responses, tissue repair, and tumor progression. M2 macrophages contribute to immune suppression and tissue remodeling in both physiological and pathological conditions [14, 15] .
2. T Cell Regulation: SPP1 modulates T cell activation and differentiation through its binding to CD44 or integrins. Notably, it suppresses the cytotoxic activity of T cells, which contributes to tumor immune evasion [16, 17, 18].
3. Dendritic Cell Activation: By enhancing dendritic cell maturation and antigen-presenting capacity, SPP1 contributes to the initiation and regulation of adaptive immune responses [10, 16].

SPP1 also plays a pivotal role in inflammation and fibrosis by promoting the recruitment and activation of inflammatory cells, as well as regulating fibroblast activity and ECM deposition. These functions make SPP1 a critical factor in the development of fibrotic diseases, such as liver fibrosis and pulmonary fibrosis [10, 19]. Moreover, SPP1 is frequently upregulated in malignant tumors, where it enhances tumor cell adhesion, migration, and immune evasion. Due to its widespread involvement in tumor progression, SPP1 is increasingly recognized as a diagnostic and prognostic biomarker in cancer [10, 20].

## SPP1 and the tumor microenvironment

### SPP1 promotes tumor stemness and therapy resistance

Cancer stem cells (CSCs) are a subset of tumor cells characterized by self-renewal capacity, high plasticity, and differentiation potential, enabling them to generate diverse progeny within the TME [21, 22]. These cells play a pivotal role in tumor initiation, progression, recurrence, and resistance to conventional therapies. Due to their ability to survive and regenerate after clinical interventions, CSCs are closely associated with tumor aggressiveness and treatment failure [23–25].

SPP1 has been identified as a critical factor in enhancing CSC stemness by promoting their self-renewal, differentiation, and survival [12]. These effects are mediated through signaling pathways activated by SPP1 binding to key receptors, particularly CD44 and integrins, which are often overexpressed in CSCs. This section explores the mechanistic interactions between SPP1, CD44, and integrins in regulating CSC behavior.

#### SPP1-CD44-Integrin axis in tumor stem cell regulation

CD44, a non-kinase transmembrane glycoprotein, serves as a well-recognized stem cell marker, expressed in embryonic stem cells, connective tissues, and bone marrow [26]. In cancer, CD44 is highly upregulated in CSCs, where it is considered a critical molecular marker for tumor initiation and progression, particularly in breast, gastric, and prostate cancers [27].

Integrins are large heterodimeric transmembrane proteins that regulate cell adhesion, migration, and survival. They possess a highly sensitive activation mechanism that responds to extracellular cues, primarily regulated through tertiary and quaternary structural changes. Within the TME, SPP1 interacts with CD44 and integrins (e.g., αvβ3 and αvβ5), activating key signaling cascades that promote CSC survival and expansion [28, 29].

#### Mechanisms by which SPP1 enhances CSC properties

**(1) Self-Renewal and Proliferation**.

- SPP1 binds to integrins (αvβ3, αvβ5) and CD44 via its RGD sequence, leading to the activation of PI3K/AKT and MAPK/ERK signaling pathways [30, 31].
- These pathways drive cell cycle progression, promote proliferation, and sustain the long-term self-renewal ability of CSCs, ensuring continuous tumor growth [32, 33].

**(2) Enhanced Adhesion and ECM Interactions**.

- SPP1 strengthens CSC adhesion to the ECM by interacting with CD44 and integrins, thereby anchoring CSCs within specialized niches [34].
- This ECM-mediated signaling supports CSC survival and helps maintain their stemness phenotype [35].

**(3) Increased Migration and Invasiveness**.

- The SPP1-CD44-Integrin axis activates focal adhesion kinase (FAK), leading to cytoskeletal reorganization and enhanced CSC motility [30, 36].
- Additionally, SPP1 stimulates the secretion of matrix metalloproteinases (MMPs), which degrade the basement membrane and ECM, significantly boosting CSC invasiveness and metastatic potential [37, 38].

**(4)Resistance to Apoptosis and Therapy**.

- SPP1 activates the PI3K/AKT pathway, which in turn regulates Bcl-2 family proteins, suppressing pro-apoptotic signals and increasing CSC survival [39, 40].
- SPP1, highly expressed in TAMs, particularly monocyte-derived subsets, is induced by granulocyte-macrophage colony-stimulating factor (GM-CSF) secreted from cancer cells under chemotherapy. This creates a feedback loop where SPP1 activates the SPP1/CD44 axis and downstream PI3K/AKT and MAPK pathways, promoting epithelial-mesenchymal transition (EMT), autophagy, aberrant glucose metabolism, and reduced drug uptake in cancer cells. Additionally, SPP1 enhances PD-L1 expression on TAMs, fostering an immunosuppressive TME that further diminishes therapeutic efficacy. The interplay between SPP1-expressing TAMs and cancer cells thus establishes a protumor niche that drives chemoresistance and poor prognosis [41].

**(5)Pro-Angiogenic Effects**.

- SPP1 promotes angiogenesis and vascular remodeling by upregulating vascular endothelial growth factor (VEGF) [42].
- This facilitates endothelial cell proliferation, migration, and increased vascular permeability, ensuring that CSCs receive an adequate nutrient and oxygen supply, further sustaining their growth and metastasis [43, 44].

### SPP1 regulates tumor-associated macrophages to promote immune suppression

SPP1 is a potent chemotactic factor for macrophages, facilitating their recruitment into the TME and reprogramming them into tumor-supportive phenotypes. High SPP1 expression in the TME is strongly correlated with increased macrophage infiltration, suggesting that SPP1 plays a direct role in guiding macrophage migration to tumor sites [12, 45, 46]. Once within the TME, SPP1 exerts profound effects on tumor-associated macrophages (TAMs), particularly by promoting their M2-like polarization, a state characterized by immune suppression, tissue remodeling, and tumor-promoting functions.

SPP1 mediates these effects by interacting with integrins (e.g., αvβ3, αvβ5) and CD44 on macrophages, activating downstream signaling pathways that enhance the expression and function of M2-associated markers. M2-polarized TAMs contribute to adaptive immune suppression, tumor angiogenesis, and metastatic progression, thereby supporting an immunosuppressive TME conducive to tumor growth [47, 48]. SPP1 achieves this by interacting with receptors on macrophages, such as integrins and CD44, activating signaling pathways that lead to the expression and function of M2 markers.

#### SPP1 + Macrophages and their role in tumor progression

SPP1 is predominantly expressed by certain macrophage subsets within the TME, many of which exhibit features of the senescence-associated secretory phenotype (SASP), further promoting tumor progression. Notably, in high-risk colorectal cancer (CRC) patients, an increased abundance of SPP1 + macrophages correlates with an immunosuppressive TME, facilitating immune evasion and metastatic dissemination [49].

Studies on head and neck squamous cell carcinoma have identified two pro-angiogenic and metastatic TAM subpopulations: SPP1 + CCL18 + and SPP1 + FOLR2 + clusters. In vitro studies indicate that SPP1 + TAMs secrete SPP1, CCL18, and CXCL8, all of which enhance tumor invasion and metastatic potential [50].

#### Key signaling pathways activated by SPP1 in TAMs

SPP1 exerts its effects on TAMs through multiple intracellular signaling pathways, each contributing to macrophage survival, immune suppression, and tumor progression:

1. PI3K/AKT Pathway: M2 Polarization and Immune Suppression.

- SPP1 enhances TAM recruitment and M2 polarization, promoting macrophage survival and resistance to apoptosis within the TME.
- M2-polarized TAMs secrete immunosuppressive cytokines, such as IL-10 and TGF-β, which inhibit anti-tumor immune responses and enable tumor cells to evade immune surveillance [47].
  (2) MAPK/ERK Pathway: TAM Expansion and Angiogenesis.

- Activation of the MAPK/ERK pathway enhances TAM proliferation, survival, and migration, leading to their progressive accumulation in the TME [51].
- These TAMs secrete VEGF and other pro-angiogenic factors, promoting tumor vascularization and sustaining the tumor’s nutrient and oxygen supply [52].
  (3) NF-κB Pathway: Inflammatory and Immune Modulation.

- The NF-κB pathway enables TAMs to secrete pro-inflammatory cytokines, which paradoxically create a chronic inflammatory state that supports tumor immune evasion [53].
- This inflammatory environment further remodels the TME in favor of tumor progression.

#### SPP1 as a defining marker of TAM Polarity

Recent studies suggest that TAM polarity can be distinguished based on CXCL9 and SPP1 expression levels, rather than the traditional M1/M2 classification. The CXCL9:SPP1 (CS) ratio in TAMs serves as a critical determinant of poor prognosis by reflecting a broader immunosuppressive and pro-tumorigenic microenvironment. A low CS ratio (SPP1-dominant) correlates with hypoxia-driven metabolic reprogramming, EMT, and diminished cytotoxic immune infiltration, fostering tumor progression and therapy resistance. Conversely, a high CS ratio (CXCL9-dominant) associates with IFN-γ-mediated immune activation and favorable outcomes. The mutually exclusive expression of these markers defines TAM polarity, with SPP1-enriched TAMs promoting immune evasion and stromal interactions that drive aggressive disease across multiple cancer types. Thus, the CS ratio encapsulates a coordinated network of tumor-permissive signals, making it a robust prognostic indicator independent of conventional M1/M2 classifications [16, 17].

### SPP1 modulates cancer-associated fibroblasts and tumor stroma

Cancer-associated fibroblasts (CAFs) are major components of the TME, playing key roles in tumors by directly interacting with malignant epithelial cells and inducing the transformation of normal fibroblasts into CAFs. SPP1 contributes to the activation and differentiation of fibroblasts into CAFs. These activated CAFs secrete various cytokines, growth factors, and MMPs, which remodel the ECM and promote tumor progression [14, 54].

Effects on CAFs:

1. CAFs Survival and Migration:

- SPP1 interacts with specific receptors on fibroblasts, such as integrins and CD44, triggering downstream signaling cascades, including PI3K/AKT and MAPK [55].
- These signaling pathways promote CAF survival, migration, and activation, allowing CAFs to infiltrate the tumor stroma and actively participate in the tumor-supporting environment.

(2) TME Remodeling and ECM Stiffness:

- SPP1 derived from tumor and immune cells activates CAFs via the CD44 signaling pathways, enhancing the secretion of collagen (COL1A1, COL1A2) and fibronectin (FN1). This extracellular matrix remodeling increases stromal stiffness and fosters a fibrotic, immune-excluded microenvironment that facilitates tumor cell migration, EMT, and therapeutic resistance [56].
- SPP1 + macrophages and POSTN + CAFs are frequently found in close proximity, and their interaction fosters ECM remodeling, helping create a tumor-promoting and immunosuppressive TME [57–59].
- Additionally, SPP1 + macrophages are highly associated with FAP + fibroblast infiltration, which correlates with abundant ECM expression and the formation of the tumor connective tissue structure, further supporting tumor growth and metastasis [60].

### SPP1 suppresses T cell-mediated anti-tumor immunity

SPP1 is currently considered an effective inhibitor of T cell activation. CD44, a receptor for SPP1, is also significantly expressed in T cells. Therefore, SPP1 can inhibit T cell activity, primarily CD8 + T cells, through its interaction with CD44 [61]. The binding of SPP1 to CD44 on T cells and tumor cells may help maintain an immunosuppressive microenvironment that supports tumor survival and growth. This interaction may involve signaling pathways that promote tumor cell survival and T cell dysfunction, leading to altered T cell responses and exhaustion, thereby reducing their efficacy against tumor cells [18, 61]. Although the specific signaling pathways of SPP1 are yet to be fully elucidated, this conclusion is inferred from observing the negative correlation between SPP1 and T cells and the restored T cell activity upon inhibiting the SPP1/CD44 pathway.

Moreover, when SPP1 influences the production of an immunosuppressive TME by CAFs, it inhibits T cell proliferation and activation, recruits regulatory T cells (Tregs) and myeloid-derived suppressor cells (MDSCs), and reduces the effectiveness of the host’s anti-tumor immune response [62, 63].

**Fig. 1:**
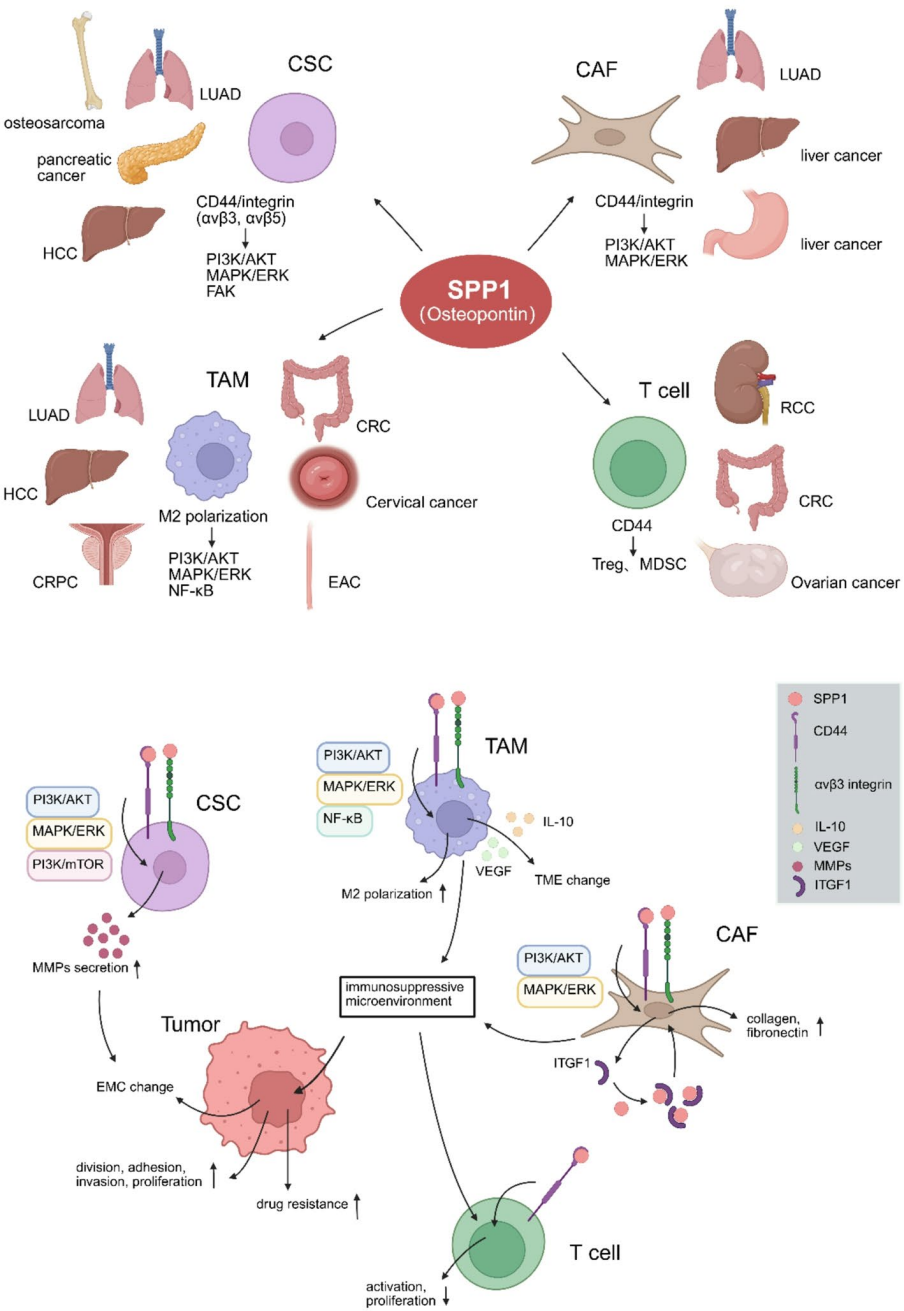
The role of SPP1 in TME cells and its impact on tumor progression. SPP1 affects TAMs and CAFs by activating their respective signaling pathways, promoting an immunosuppressive microenvironment, which reduces T cell activity and proliferation. SPP1 also influences CSCs, increasing MMP secretion and driving changes in the tumor extracellular matrix

In summary, SPP1 interacts with CD44 and integrins (αvβ3, αvβ5, ITGB1) through its RGD motif, orchestrating the activation of PI3K/AKT, MAPK/ERK, and FAK signaling cascades. In TAMs, CAFs, and CSCs, SPP1–CD44/integrin engagement activates FAK–PI3K–AKT and ERK pathways to promote activation and survival, whereas in T cells, SPP1 mainly engages CD44s and α4β1 integrins to induce inhibitory feedback and T cell exhaustion [34]. These convergent pathways collectively enhance the proliferative, self-renewal, migratory, and invasive capacities of cancer stem cells and other tumor-associated cells, while reinforcing ECM adhesion and fostering an immunosuppressive tumor microenvironment. Through PI3K/AKT, SPP1 modulates Bcl-2 family proteins, promotes autophagy, and confers chemoresistance. In cancer-associated fibroblasts and tumor-associated macrophages, this same CD44/integrin–PI3K/AKT axis sustains cell survival, motility, and immune regulatory functions, underscoring its central role in tumor progression and therapeutic resistance (Fig. 1).

## OPN in TME: examples from different types of cancer

### Osteosarcoma

Denhardt et al. first discovered SPP1’s role in osteosarcoma through in vitro studies, revealing that elevated SPP1 levels boost tumor cell invasion and migration via interaction with integrin αvβ3 [64]. Liu et al. later confirmed this by showing that SPP1 increases MMP-2 and MMP-9 levels, further enhancing osteosarcoma cell invasiveness [65]. Additionally, Berge et al. found that SPP1 is a key downstream effector in S100A4-driven osteosarcoma cell invasion and metastasis, supported by both lab and animal studies [66]. Conversely, Dalla-Torre et al. used quantitative PCR on patient samples and identified SPP1 as a differentially expressed gene, linking it more to inflammation and bone remodeling than directly to tumor progression [67]. Their study also observed increased LAMP3 expression in osteosarcoma cases with lung metastasis. Li et al. found that knocking out LAMP3 increased SPP1 expression, reducing cell invasiveness. However, knocking out both SPP1 and LAMP3 reversed this effect, suggesting that SPP1 may act downstream of LAMP3 and protect against metastasis [68]. In summary, although SPP1 is not essential for the development of osteosarcoma, further research is necessary to elucidate its underlying mechanisms.

### Liver cancer

Pan et al. first reported the role of SPP1 in hepatocellular carcinoma (HCC), finding it highly expressed and linked to greater tumor aggressiveness, early recurrence, and poor prognosis [69]. Kim J confirmed these findings with elevated plasma levels of SPP1 in HCC patients [70]. El-Tanani MK’s team further investigated SPP1’s involvement in HCC metastasis, connecting it to cell signaling and metastatic behavior [71].

SPP1 contributes to HCC development through several mechanisms:

Effects on Tumor Cells: Liu et al. confirmed SPP1 expression in HCC SK cells via immunofluorescence staining. Reducing SPP1 with siRNA decreased cell migration and colony formation, suggesting an oncogenic role [14]. Furthermore, Wang et al. found that HHLA2 enhances liver cancer cell growth and invasion through the SPP1/PI3K/AKT pathway, and lowering SPP1 levels can counteract HHLA2’s effects [72].

Effects on TME: HCC tumors are composed of malignant cells, CAFs, and TAMs. SPP1 is mainly found in malignant cells and TAMs. Liu et al. observed colocalization of SPP1 and CD68 in HCC tissues. Knocking out SPP1 in SK cells and co-culturing them with M0 macrophages for 72 h led to a downregulation of M2 markers, indicating that SPP1 contributes to M2 polarization [14]. Liu et al. found that blocking or deleting SPP1 in macrophages disrupts the tumor immune barrier, increases cytotoxic T cell infiltration, and improves anti-PD-1 therapy effectiveness in a mouse liver cancer model [54]. Zhang et al. identified a unique interaction between macrophages and malignant cells through the SPP1-CD44 axis by analyzing single-cell transcriptomes from HCC patients [73].

### Colorectal cancer

Klement et al. reported that SPP1 plays a significant role in CRC. Their clinical studies showed that elevated SPP1 levels in the TME are linked to tumor progression by suppressing CD8 + T cell proliferation and IFN-γ production [18]. Later, Amilca-Seba et al. confirmed the role of SPP1 in CRC through in vitro and in vivo experiments. They discovered that the Slug(Snail family transcriptional repressor 2, SNAI2) transcription factor promotes tumor invasiveness and metastasis by directly regulating SPP1 expression [74].

Current understanding of SPP1’s role in CRC highlights several key mechanisms:

Tumor Growth and Metastasis: Xu et al. found that SPP1 mRNA and protein levels are significantly higher in CRC tissues than in non-cancerous ones, promoting metastasis by activating EMT [75].

TME and Immune Evasion: Qi et al. observed that FAP + fibroblasts and SPP1 + macrophages negatively correlate with lymphocyte infiltration. These cells modify the tumor environment, enhancing growth and spread [76].

Prognostic Implications: Choe EK et al. showed that increased SPP1 levels in cancer tissues correlate with lower survival rates and a worse prognosis for CRC patients [77, 78].

### Cervical cancer

Numerous studies confirm SPP1’s role in cervical cancer. A TCGA and GEO database study found that high SPP1 expression correlates with poor overall survival, indicating a poor prognosis. This study also revealed SPP1’s link to immune cell infiltration, especially neutrophils, macrophages, and dendritic cells [79]. In subsequent research, Qin et al. were the first to clinically highlight SPP1’s importance, noting its higher expression in cervical cancer tissues and its association with age, FIGO stage, tumor size, lymphovascular invasion, and prognosis [80]. Additionally, Chen et al. discovered that inhibiting SPP1 reduces HeLa cell growth, induces apoptosis, and increases cisplatin sensitivity by downregulating the PI3K/AKT pathway [81]. Poleboyina PK identified SPP1 as highly upregulated in cervical cancer and noted that entrectinib, an SPP1 inhibitor, effectively slows cancer progression [82].

### Lung adenocarcinoma

Li B, Yi X and Tang H found that SPP1 levels are much higher in lung adenocarcinoma (LUAD) tissues and cells compared to normal ones [46, 83, 84]. SPP1 expression is positively linked to TNM stage, lymph node metastasis, and invasion depth. Studies both in vitro and in vivo demonstrated that lowering SPP1 levels can reduce cell migration and invasion, and patients with elevated SPP1 levels tend to have lower survival rates. This pattern was not seen in lung squamous cell carcinoma. Thus, SPP1 is a reliable marker for immune invasion status and prognosis in LUAD. Furthermore, various studies have highlighted SPP1 as a key indicator of poor prognosis in LUAD, showing that increased SPP1 levels contribute to immune evasion, metastasis, and drug resistance [41, 45, 84–86].

Current insights into SPP1’s role in lung cancer highlight its impact on the TME. Monocytes and macrophages interact with tissue stem cells and NK cells via the SPP1 pathway, while stem cells communicate with T and B cells through the CXCL pathway. Key genes in the SPP1 pathway are negatively correlated with CD4 + and CD8 + T cells, and SPP1 expression in LUAD tumors is linked to poorer prognosis. Conversely, CXCL12, part of the CXCL pathway, positively correlates with CD4 + and CD8 + T cells, counteracting SPP1’s effects in LUAD [87]. Additionally, SPP1 promotes immune evasion by increasing PD-L1 levels, which enhances macrophage polarization [41, 48, 86]. Regarding tumor cells, the SPP1/CD44 axis is crucial for communication between tumor cells and TAMs [41]. SPP1 influences the expression of collagen type XI alpha 1 (COL11A1), and reducing SPP1 significantly lowers COL11A1 levels. Importantly, the reduction in cell migration and invasion, along with changes in EMT marker expression due to SPP1 reduction, can be reversed by overexpressing COL11A1^46^.

### Esophageal cancer

Thomas et al. first identified that SPP1 is overexpressed in esophageal adenocarcinoma (EAC), emphasizing its role in enhancing tumor cell invasion and spread. They observed significantly higher SPP1 levels in EAC patients compared to those with Barrett’s esophagus and low-grade dysplasia [88]. Song et al. confirmed these results using bioinformatics and lab experiments, revealing that SPP1 is also highly expressed in esophageal squamous cell carcinoma (ESCC), where it aids tumor cell migration and invasion by interacting with MMPs [89]. Recent studies indicate that SPP1 contributes to tumor growth through multiple pathways.

Effects on the TME: SPP1 activates the CD44/PI3K/AKT pathway, attracting macrophages and causing them to polarize to the M3 type. This action increases the expression of VEGFA and IL6, promoting the advancement of ESCC [52].

Effects on Tumor Cells: Elevated SPP1 levels are associated with radioresistance in esophageal cancer. SPP1 enhances the repair of DNA damage through the JAK2/STAT3 pathway, boosting cell survival and leading to radiotherapy resistance [90].

### Ovarian cancer

Initial studies on SPP1 in ovarian cancer involved analyzing gene expression data across various cancers. Researchers discovered that SPP1 levels were significantly higher in several tumors, including ovarian cancer, indicating its potential role in tumor progression. These studies utilized immunohistochemistry and gene expression databases to show SPP1’s widespread upregulation. Later research explored SPP1’s specific mechanisms in ovarian cancer. One study confirmed high SPP1 expression in ovarian cancer cells through immunohistochemistry and examined its link to tumor-infiltrating immune cells. The findings revealed that SPP1 is not only highly expressed in tumor cells but also closely associated with immune cell infiltration and regulation of the TME [91].

Current insights into SPP1’s role in ovarian cancer highlight its impact on tumor cells and the TME.

Effects on Tumor Cells: In epithelial ovarian cancer, SPP1 expression is elevated, enhancing cancer cell migration, invasion, and growth. It also influences the ITGB1/FAK/AKT pathway, with miR-181a regulating SPP1 via its 3’ UTR [36].

Effects on the TME: Immune cells in the TME are crucial for tumor progression and affect patient outcomes. In ovarian cancer, SPP1 expression is positively linked to the presence of CD4 + T cells, CD8 + T cells, macrophages, neutrophils, and dendritic cells. Notably, dendritic cell infiltration and SPP1 expression are key factors in ovarian cancer prognosis [91].

Prognostic Implications: High SPP1 levels are associated with poorer outcomes, including reduced disease-free and overall survival. SPP1 may also worsen prognosis through ITGA5-related pathways [91].

### Prostate cancer

Chandran identified SPP1 as a gene linked to prostate cancer metastasis through gene expression profiling. Increased SPP1 levels are associated with greater cell adhesion, migration, and invasion, suggesting its role in cancer spread [92]. Another study found that SPP1, produced by macrophages in the tumor environment, stimulates the growth of prostatic intraepithelial neoplasia (PIN) cells via the AKT and JNK pathways. Using 3D cultures and in vivo experiments, it was shown that macrophage-derived SPP1 speeds up the transition from PIN to prostate cancer [93]. Recently, Pang et al. highlighted SPP1’s role in drug resistance, especially to enzalutamide in Castration-Resistant Prostate Cancer (CRPC). Their research demonstrated that SPP1 supports cancer cell survival during androgen deprivation therapy through the PI3K/AKT and ERK1/2 pathways, as seen in patient-derived organoids and CRPC cell lines. Furthermore, prostate cancer development is linked to increased macrophage infiltration [94].​.

SPP1 is crucial for prognosis in prostate cancer, with high expression levels linked to worse clinical outcomes. It regulates immune cell infiltration, particularly macrophages and T cells, contributing to an immunosuppressive tumor environment. This may lead to disease progression and resistance to therapy. Thus, SPP1 is seen as both a prognostic marker and a potential therapeutic target.

### Pancreatic cancer

Ouyang et al. demonstrated SPP1’s role in promoting pancreatic cancer through in vitro and in vivo experiments. They showed that FOXD1-AS1 regulates miR-570-3p, increasing SPP1 levels and supporting the growth of CSCs [95]. Yang et al. confirmed SPP1’s impact in pancreatic ductal adenocarcinoma, finding that LINC01133 stabilizes SPP1 mRNA, boosting cell growth, movement, and survival [96]. Another study found that SPP1 helps cancer cells evade the immune system via the WDR5-H3K4me3 pathway [12]. Further research indicates the interaction between SPP1 and CD44 is a key mechanism in maintaining cancer stem cell characteristics and enhancing chemotherapy resistance [97].

### Renal cell carcinoma

Rabjerg M reported that high levels of SPP1 are linked to poor outcomes in clear cell renal cell carcinoma (ccRCC). They showed that increased SPP1 promotes tumor growth and prevents cell death by activating the NF-κB pathway [98]. SPP1 interacts with receptors like CD44 or integrin αvβ3, initiating pathways that include NF-κB. This activation boosts anti-apoptotic genes, such as Bcl-2 and the IAP family, thereby blocking apoptosis. Moreover, NF-κB activation can further elevate SPP1 levels, creating a feedback loop that aids tumor survival and growth. Wang Y confirmed these results through immunofluorescence studies, revealing that the YBX1 protein regulates SPP1 via its interaction with G3BP1. YBX1, known for its role in various cancers, and G3BP1, an RNA-binding protein often found in tumors, form a complex in renal cell carcinoma that activates SPP1 and the NF-κB pathway. This pathway is crucial for cell growth, survival, and inflammation, with its abnormal activation linked to cancer progression and resistance to treatment [99]. Additionally, Zhang et al. using single-cell RNA sequencing, identified that SPP1 is highly expressed in ccRCC. They found that SPP1 is secreted by certain CSCs in ccRCC, promoting disease progression by interacting with integrin receptors and activating pathways like ILK and JAK/STAT [100].

**Fig. 2:**
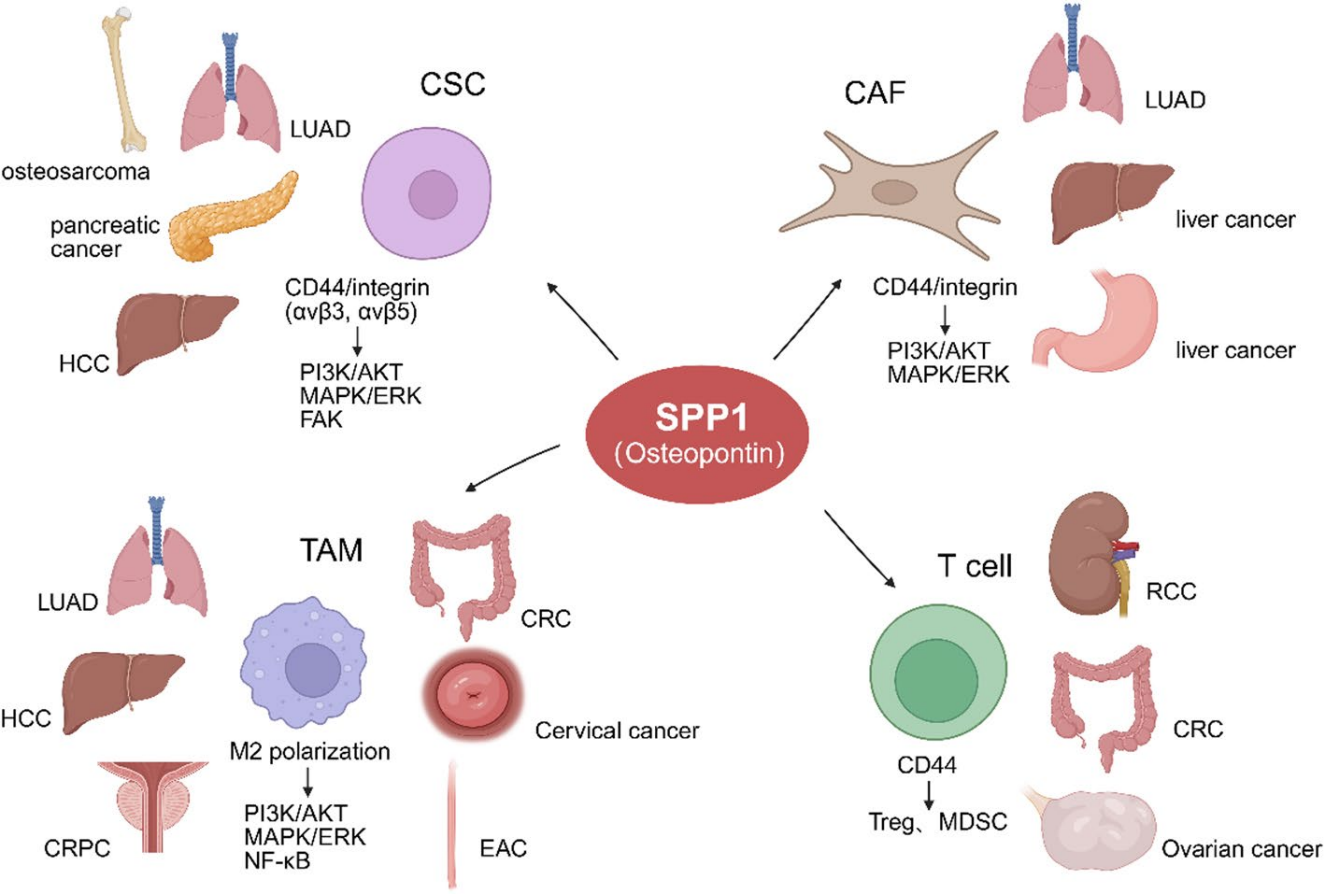
An integrated overview of the context-dependent functions of SPP1 within the TME across multiple cancer types. SPP1 exerts differential regulatory effects on key cellular components—including CSCs, CAFs, TAMs, and T cells—predominantly through interactions with CD44 and integrin receptors. These interactions activate canonical signaling cascades such as PI3K/AKT, MAPK/ERK, FAK, and NF-κB, thereby promoting tumor progression, immune modulation, and microenvironmental remodeling. The figure also delineates the predominant TME component engaged by SPP1 in distinct malignancies, underscoring the heterogeneity of SPP1-mediated signaling across tumor contexts

The roles of SPP1 across various tumor types are systematically summarized in a table (Table 1), and a schematic figure was generated to depict the major TME components engaged by SPP1 in different malignancies (Fig. 2).

**Table 1:** SPP1 expression and its role in various tumors

Tumor Type	Research Methods	SPP1 Expression Level and its function	Role of SPP1 in Tumor	References
Osteosarcoma	In vitro studies, in vivo models, real-time quantitative PCR	High, facilitating tumor invasion and metastasis	Enhances invasion and migration via integrin αvβ3, upregulates MMP-2 and MMP-9, acts as downstream effector in S100A4-mediated invasion, associated with inflammatory responses and bone remodeling	[58, 59, 60, 61, 62]
Liver cancer	Clinical studies, immunofluorescence staining, cell culture, mouse models, single-cell transcriptomes	High, facilitating tumor growth and invasion	Linked to tumor aggressiveness, early recurrence, poor prognosis, enhances cell migration and colony formation, involved in PI3K/AKT pathway, affects tumor microenvironment and immune response	[11, 48, 63, 64, 65, 66, 67]
Colorectal cancer	Clinical studies, in vitro and in vivo experiments	High, facilitating tumor growth and metastasis	Suppresses CD8 + T cell proliferation, inhibits IFN-γ production, promotes invasiveness and metastasis via Slug/SNAI2, activates EMT, affects tumor microenvironment and immune evasion, correlates with poor prognosis	[15, 68, 69, 70, 71, 72]
Cervical cancer	TCGA and GEO database analysis, clinical studies, in vitro experiments	High, facilitating tumor invasion and therapeutic resistance	Correlates with poor prognosis, linked to immune cell infiltration, associated with clinical features (age, FIGO stage, etc.), reduces cell growth and increases cisplatin sensitivity, inhibited by entrectinib	[73, 74, 75, 76]
Lung adenocarcinoma	Tissue and cell analysis, in vitro and in vivo studies	High, facilitating tumor invasion, metastasis and therapeutic resistance	Linked to TNM stage, lymph node metastasis, invasion depth, immune evasion, metastasis, drug resistance, poor prognosis, affects tumor microenvironment, promotes PD-L1 levels, influences COL11A1	[35, 39, 40, 42, 77, 78, 79, 80, 81]
Esophageal Adenocarcinoma	Clinical observations, bioinformatics, lab experiments	High, facilitating tumor invasion and metastasis	Enhances tumor cell invasion and spread, higher levels compared (ESCC)	[82]
Esophageal Squamous Cell Carcinoma	Bioinformatics, lab experiments	High, facilitating tumor therapeutic resistance	Aids tumor cell migration and invasion via MMPs, activates CD44/PI3K/AKT pathway, promotes macrophage polarization, increases VEGFA and IL6 secretion, contributes to radioresistance through JAK2/STAT3 pathway	[46, 83, 84]
Ovarian cancer	Gene expression analysis, immunohistochemistry, lab experiments	High, facilitating tumor invasion and metastasis	Enhances cancer cell migration, invasion, and growth via ITGB1/FAK/AKT pathway; regulated by miR-181a; associated with immune cell infiltration (CD4 + T cells, CD8 + T cells, macrophages, neutrophils, dendritic cells); impacts tumor microenvironment and prognosis (linked to reduced survival)	[30, 85]
Prostate cancer	Gene expression profiling, 3D cultures, in vivo experiments, patient-derived organoids	High, facilitating tumor invasion, metastasis and therapeutic resistance	Enhances cell adhesion, migration, and invasion; stimulates growth of PIN cells via AKT and JNK pathways; accelerates transition from PIN to prostate cancer; supports CRPC cell survival during androgen deprivation therapy (PI3K/AKT and ERK1/2 pathways); linked to drug resistance and immune cell infiltration (especially macrophages and T cells)	[86, 87, 88]
Pancreatic cancer	In vitro and in vivo experiments	High, facilitating tumor growth and therapeutic resistance	Regulated by FOXD1-AS1/miR-570-3p; supports cancer stem cell growth; stabilized by LINC01133; boosts cell growth, movement, survival; aids immune evasion via WDR5-H3K4me3 pathway; interacts with CD44 to maintain stem cell traits and enhance chemotherapy resistance	[9, 89, 90, 91]
Renal cell carcinoma	Immunofluorescence, single-cell RNA sequencing	High, facilitating tumor growth and therapeutic resistance	Promotes tumor growth and prevents cell death via NF-κB pathway; interacts with CD44/integrin αvβ3; boosts anti-apoptotic genes; regulated by YBX1 and G3BP1; secreted by cancer stem cells, activating ILK and JAK/STAT pathways	[92, , 94]

SPP1 expression and its role in various Tumors. This table summarizes the role of SPP1 in various tumor types based on findings from clinical studies, in vitro and in vivo experiments, and bioinformatics analyses. SPP1 is highly expressed across multiple cancers and is associated with tumor progression, poor prognosis, immune evasion, metastasis, drug resistance, and alterations in the tumor microenvironment. It interacts with various receptors and signaling pathways, such as integrin αvβ3, PI3K/AKT, NF-κB, JAK/STAT, and ITGB1/FAK/AKT, promoting cell growth, migration, invasion, and survival. Additionally, SPP1 influences immune cell infiltration and inflammatory responses, contributing to tumor aggressiveness and therapy resistance. Specific roles and mechanisms of SPP1 in each tumor type are detailed, with references provided for further exploration

## SPP1 as a prognostic biomarker in cancer

SPP1 expression is differentially altered in many malignancies and can serve as a potential prognostic biomarker. However, the relationship between SPP1 expression and prognosis is not straightforward. Some studies have reported that decreased SPP1 expression is a poor prognostic factor for CRC and endometrial cancer [101, 102]. For example, Collins et al. found that higher SPP1 expression is beneficial, being associated with improved prognosis in patients with pancreatic cancer [103]. This study, through large-scale clinical data, rigorous statistical analysis, and cross-cancer type comparisons, was the first to propose the protective role of OPN in pancreatic cancer. The authors proposed that the protective role of OPN may be linked to the unique tumor microenvironment of pancreatic cancer. OPN may modulate the crosstalk between cancer cells and the stroma, thereby suppressing the aggressive phenotype of tumors. Conversely, several other studies have highlighted that high levels of SPP1 are associated with poor prognosis in many solid malignancies, such as prostate, lung, stomach, and breast cancers [104–107]. For instance, Wong et al. demonstrated that in bladder cancer patients with high tumor grade, overexpression of SPP1 is significantly associated with shortened patient survival [108]. Therefore, SPP1 may serve as a valuable prognostic biomarker and a potential target for cancer therapy in various solid tumors.

Compared with PD-L1, a canonical immune checkpoint molecule mainly expressed on tumor and immune cells that directly inhibits effector T cell activity, SPP1 functions as an upstream or indirect immune suppressor. It can upregulate PD-L1 expression, drive TAM polarization, and promote T cell exhaustion, thereby synergizing with the PD-1/PD-L1 axis in shaping the immunosuppressive microenvironment [48, 109].

In contrast to VEGF, which directly drives angiogenesis and serves as a validated therapeutic target, SPP1 indirectly promotes vascularization and stromal remodeling through the induction of VEGF, MMPs, and pro-inflammatory signaling pathways. This cooperation supports hypoxic and pro-metastatic niches within the tumor microenvironment [110].

Regarding CAFs (e.g., α-SMA⁺ or FAP⁺ fibroblasts), SPP1 is secreted by certain CAF subsets or induced by CAF-derived factors, mediating cross-talk among CAFs, tumor cells, and macrophages to promote invasion, immune suppression, and therapeutic resistance [111].

Among TAM markers (e.g., CD68, CD163), SPP1 is highly expressed in a specific TAM subset (SPP1⁺ TAMs) associated with hypoxia, pro-metastatic signaling, and T cell dysfunction. Therefore, SPP1 serves not only as a functional mediator but also as a molecular marker identifying tumor-promoting macrophage populations [112].

## Targeting SPP1: potential therapeutic strategies

Given the critical role of SPP1 in promoting tumor invasiveness and poor prognosis, targeting SPP1 with inhibitors or antibodies might offer therapeutic benefits. This strategy could involve blocking the interaction between SPP1 and its receptors or inhibiting its downstream signaling pathways to counteract its pro-tumor effect. Several potential therapeutic strategies are outlined below:

### Direct inhibition of SPP1

Given the pivotal role of SPP1 in promoting tumor progression through multiple mechanisms, directly targeting SPP1 itself represents a promising therapeutic strategy. Studies have demonstrated that BET inhibitors, such as NHWD-870, JQ-1, and BMS-986,158, suppress SPP1 expression by targeting the BRD4–NF-κB signaling axis. Specifically, BET inhibition downregulates BRD4, which normally activates NF-κB transcription via promoter binding, leading to reduced NF-κB expression. As NF-κB binds to the SPP1 promoter to enhance its transcription, diminished NF-κB levels attenuate promoter occupancy, thereby lowering SPP1 transcriptional activity [113]. This constitutes one of the rare documented instances where *SPP1* expression is downregulated via targeting its upstream regulatory axis. However, the efficacy of BET inhibitors is highly dependent on tumor-specific molecular features, such as BRD4 expression levels and NF-κB pathway activity, which may lead to interpatient variability in therapeutic response.

### Targeting the CD44/SPP1 and integrin/SPP1 axes

Targeting the CD44/SPP1 and integrin/SPP1 axes represents a promising strategy for cancer therapy, as these interactions play critical roles in the TME. By interacting with tumor-associated cells such as fibroblasts and macrophages, SPP1 activates signaling pathways like PI3K/AKT and MAPK, promoting cell survival, migration, and invasion [30]. Additionally, through binding with CD44 and integrins, SPP1 enhances ECM remodeling, increases tumor stroma stiffness, and supports immune evasion [13, 54]. By targeting these axes, it may effectively inhibit tumor cell growth and metastasis, improve anti-tumor immune responses, and provide new therapeutic approaches.

However, CD44 and integrins (e.g., αvβ3, αvβ5) are broadly expressed in normal tissues, including immune and epithelial cells. SPP1 regulates bone remodeling, immune cell recruitment, and tissue repair, while CD44 mediates hematopoietic stem cell homing, lymphocyte activation, and epithelial barrier maintenance. Evidence from knockout and antibody-based studies indicates that long-term or systemic inhibition of SPP1/CD44 may lead to impaired wound healing, altered bone mineralization, immune dysregulation, and delayed tissue regeneration [114–116]. Moreover, SPP1 exerts its functions through multiple signaling cascades, including PI3K/AKT and MAPK. Single-pathway inhibition may be limited by compensatory activation of alternative routes.

### Targeting the PD-L1-SPP1 axis

Immune checkpoint inhibitors targeting PD-1 or PD-L1 have significantly improved outcomes for patients with various types of cancer. PD-L1, quantified by immunohistochemistry, is currently the most validated, used, and accepted biomarker to guide the selection of patients for anti-PD-1 or anti-PD-L1 antibody therapy [117]. Studies have found a close association between the reduction of PD-L1 and SPP1, indicating that they can mutually enhance therapeutic effects [54]. Another study revealed that *SPP1*⁺ macrophages—particularly the subset expressing signal regulatory protein α (SIRPα)—exhibit high PD-L1 expression across multiple tumor types, suggesting that these macrophages may contribute to immune regulation through PD-L1/PD-1 interactions [118]. Additionally, inhibiting PD-L1 can induce a decrease in M2 markers (IL-10 and Arg-1) and an increase in M1 markers (IL-12 and TNF-α), suggesting an enhanced anti-tumor immune response [48]. Therefore, PD-L1 is a viable target for cancer therapy. Available targeting drugs include pembrolizumab, atezolizumab, and bevacizumab [117].

Although PD-L1 inhibitors (e.g., pembrolizumab) are approved, the mechanistic interplay between SPP1 and PD-L1 remains incompletely understood. In patients with high SPP1 expression, therapeutic efficacy may be influenced by TAM polarization states and T cell exhaustion. Additionally, the correlation between SPP1 and PD-L1 expression requires validation in large-scale clinical trials, and no standardized assays or threshold values currently exist.

### Targeting the CXCL9/SPP1 axis

The CXCL9/SPP1 axis has significant prognostic implications in cancer, and targeting factors that influence this axis may provide therapeutic benefits. Local environmental signals such as IFN-γ and hypoxia can respectively promote the expression of CXCL9 and SPP1, thereby affecting the polarity of TAMs. In various tumors, CXCL9 and SPP1 expressions are generally negatively correlated. Enhancing IFN-γ to promote CXCL9 expression can inhibit SPP1 expression [16]. Moreover, IFN-γ can bind to interferon gamma receptors (IFNGR), regulating the JAK-STAT pathway. This interaction activates antigen-presenting cells and promotes the differentiation of type I helper T cells (Th1 cells) by upregulating the transcription factor T-bet. This process achieves anti-tumor effects and enhances therapeutic efficacy [119]. Using IFN-γ drugs to increase IFN-γ concentration in the body is a viable method to enhance the anti-tumor immune response and inhibit SPP1 expression. This approach can potentially shift the TME towards one that supports immune-mediated tumor destruction. While IFN-γ can upregulate CXCL9 and suppress SPP1, systemic administration may induce fever, fatigue, and other toxicities, and maintaining effective intratumoral concentrations remains difficult.

### Targeting the VEGFA-MMP9 axis

A study on HCC found that activation of the VEGFA signaling pathway might promote the secretion of MMP9 by SPP1-positive TAMs. MMP9 plays a significant role in the ECM, as it can degrade the ECM, thereby promoting tumor invasion and metastasis [37, 38]. Therefore, targeting VEGFA is a viable therapeutic option. Available targeting drugs include Surufatinib, Nintedanib, and Sorafenib, all of which have been approved and have shown certain efficacy [120–122].

**Fig. 3:**
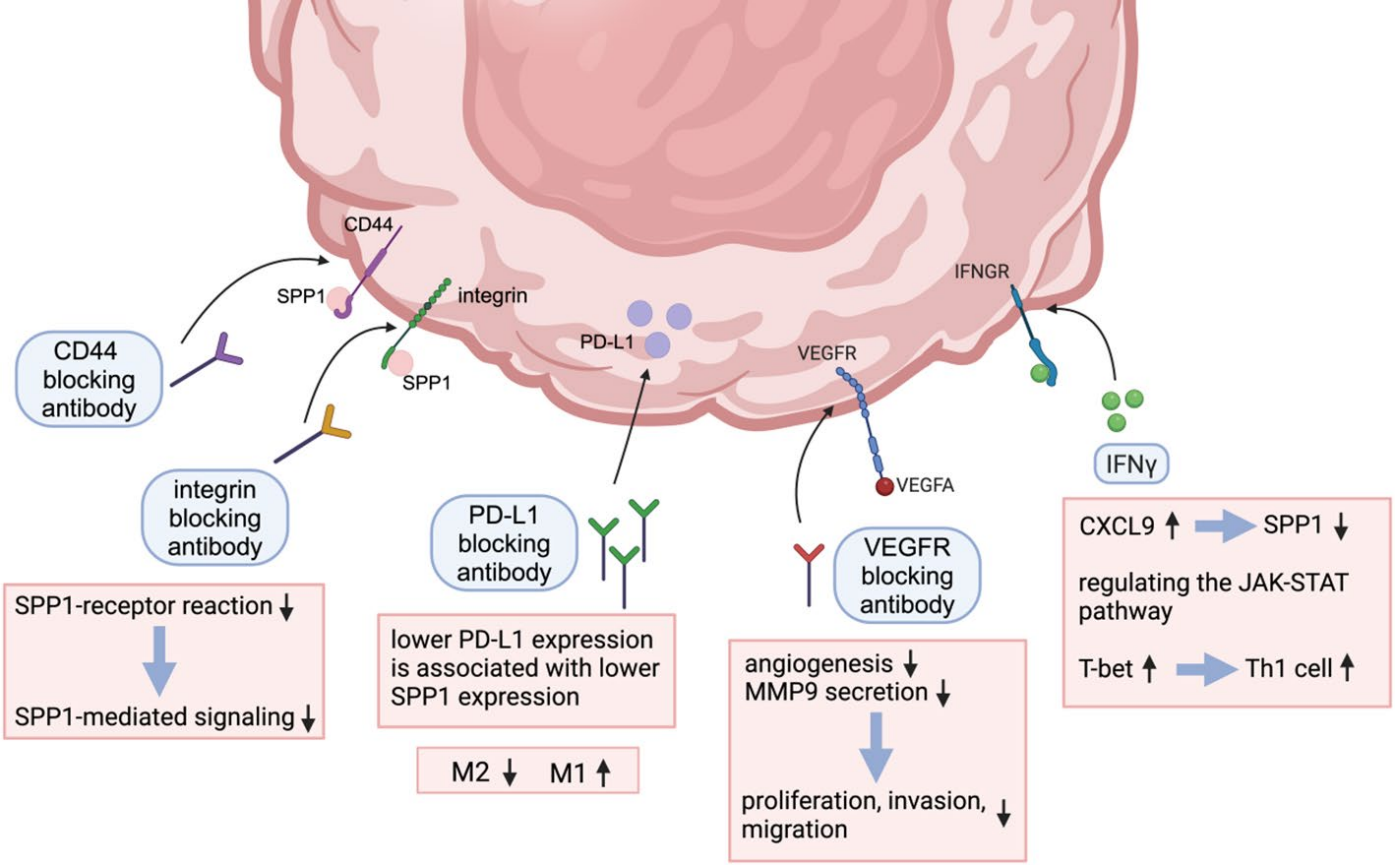
Mechanisms of immune modulation through SPP1-receptor interactions in the tumor microenvironment. Blocking antibodies targeting CD44, integrin, PD-L1, and VEGFR impact SPP1-mediated signaling pathways. Inhibition of CD44 or integrin reduces SPP1-receptor interactions, leading to decreased SPP1 signaling. Lower PD-L1 expression correlates with reduced SPP1 expression, resulting in a shift from M2 to M1 macrophage polarization. VEGFR blockade decreases angiogenesis and MMP9 secretion, limiting tumor proliferation, invasion, and migration. Increased IFN-γ enhances CXCL9 expression, which suppresses SPP1 expression and modulates the JAK-STAT signaling pathway. Additionally, IFN-γ upregulates T-bet, thereby promoting Th1 cell differentiation

Collectively, the inhibitory strategies depicted in Fig. 3 target critical SPP1-mediated receptor interactions and downstream signaling cascades—most notably the CD44/integrin-PI3K/AKT axis, PD-L1–SPP1 feedback loop, CXCL9/SPP1 polarity regulation, and VEGFA–MMP9 pathway (Fig. 3). By disrupting these molecular circuits, such interventions have the potential to attenuate tumor cell proliferation, invasion, angiogenesis, and immune evasion, thereby reprogramming the tumor microenvironment toward an anti-tumor phenotype.

## Summary and future directions

SPP1 plays a critical role in the initiation and progression of various cancers. Through interactions with multiple receptors, SPP1 activates key cancer-related signaling pathways that promote cell survival, proliferation, migration, and invasion. It also contributes to the recruitment of immune cells into the TME, thereby facilitating immunosuppressive conditions that support tumor growth.

Aberrant upregulation of SPP1 is observed across multiple malignancies, where it critically shapes the immunosuppressive landscape of the TME through modulation of macrophage polarization. Evidence indicates that SPP1 promotes the skewing of macrophages toward the M2 phenotype, which is closely associated with anti-inflammatory responses, tissue remodeling, and tumor progression. In hepatocellular carcinoma, SPP1 activates the PI3K/AKT and MAPK/ERK signaling pathways, thereby enhancing the recruitment and functional activity of M2-like TAMs, suppressing CD8⁺ T-cell cytotoxicity, and facilitating immune evasion. Similarly, in lung and colorectal cancers, elevated SPP1 expression correlates with the enrichment of M2-like TAMs, which secrete immunosuppressive cytokines such as IL-10 and TGF-β, further dampening antitumor immunity. Moreover, interactions between SPP1⁺ macrophages and CAFs exacerbate ECM stiffening, providing a physical scaffold that supports tumor growth and metastasis. Notably, SPP1 expression inversely correlates with that of CXCL9, giving rise to a novel macrophage polarization index (CXCL9:SPP1 ratio), in which a lower ratio predicts poorer prognosis and a more immunosuppressive TME. Collectively, these findings underscore the pivotal role of SPP1 in orchestrating a tumor-promoting immune milieu, highlighting its potential as a therapeutic target in cancer immunotherapy.

However, SPP1’s role in cancer is complex, as it can also exhibit a protective function in certain tumor types, acting as a “double-edged sword”. This dual role means that the impact of SPP1 in different cancers remains not fully understood.

At present, there are no specific drugs targeting SPP1 for cancer treatment, highlighting an unmet need. Developing inhibitors or activators of SPP1 could open new avenues for cancer therapy. Given the complexity of the TME and the variety of tumor signaling pathways, a promising strategy might involve developing targeted therapies based on the specific tumor-promoting mechanisms driven by SPP1.

However, even if highly specific therapeutic agents targeting SPP1 become available in the future, several challenges will remain. For instance, although SPP1 is abundantly expressed in the tumor stroma, antibodies or small-molecule inhibitors may fail to achieve adequate intratumoral penetration due to ECM barriers or aberrant vascular architecture. Given that CAFs play a pivotal role in establishing the stromal framework, a combinatorial approach employing CAF inhibitors, VEGF inhibitors, or MMPs could be considered to attenuate these physical barriers, thereby enhancing the therapeutic efficacy of SPP1-targeted agents. In addition, it is noteworthy that SPP1 does not universally exert tumor-promoting effects across all malignancies. For instance, in pancreatic cancer, elevated SPP1 expression has been correlated with improved patient prognosis. The underlying mechanisms appear to differ depending on the biological context. In tumor-promoting settings, SPP1 interacts with its receptors—primarily integrins and CD44—to activate multiple downstream signaling cascades that facilitate cancer cell migration, invasion, and metastatic spread. Conversely, in tumor-suppressive contexts, SPP1 may contribute to the maintenance of stromal homeostasis, thereby impeding the dissemination of malignant cells. The precise determinants of this functional duality remain largely undefined and merit further investigation. Consequently, even in the event that highly specific SPP1-targeted therapeutics become available, their clinical application will necessitate the development of precision treatment strategies that are tailored to the particular cancer type, molecular context, and stage of disease progression.

In conclusion, while SPP1 holds significant promise as a therapeutic target, current research remains limited. Future studies should focus on large-scale clinical trials and more detailed exploration of cellular mechanisms to better understand and harness SPP1’s potential in cancer treatment.

## Data Availability

No datasets were generated or analysed during the current study.

## References

[1] Oldberg A, Franzen A, Heinegard D. Cloning and sequence analysis of rat bone sialoprotein (osteopontin) cDNA reveals an Arg-Gly-Asp cell-binding sequence. Proc Natl Acad Sci U S A. 1986;83(23):8819–23. 3024151 10.1073/pnas.83.23.8819 PMC387024

[2] T Butler W. The nature and significance of osteopontin. Connect Tissue Res. 1989;23(2–3):123–36. 2698313 10.3109/03008208909002412

[3] Lin EY, Xi W, Aggarwal N, Shinohara ML. Osteopontin (OPN)/SPP1: from its biochemistry to biological functions in the innate immune system and the central nervous system (CNS). Int Immunol. 2023;35(4):171–80. 36525591 10.1093/intimm/dxac060 PMC10071791

[4] Kaleta B. The role of osteopontin in kidney diseases. Inflamm Res. 2019;68(2):93–102. 30456594 10.1007/s00011-018-1200-5

[5] Clemente N, Raineri D, Cappellano G, Boggio E, Favero F, Soluri MF, Dianzani C, Comi C, Dianzani U, Chiocchetti A. Osteopontin Bridging Innate and Adaptive Immunity in Autoimmune Diseases. *J Immunol Res* 2016, *2016*, 7675437. 10.1155/2016/7675437 PMC5206443 28097158

[6] Lok ZSY, Lyle AN. Osteopontin in vascular disease. Arterioscler Thromb Vasc Biol. 2019;39(4):613–22. 30727754 10.1161/ATVBAHA.118.311577 PMC6436981

[7] Shao Z, Morser J, Leung LLK. Thrombin cleavage of osteopontin disrupts a pro-chemotactic sequence for dendritic cells, which is compensated by the release of its pro-chemotactic C-terminal fragment. J Biol Chem. 2014;289(39):27146–58. 25112870 10.1074/jbc.M114.572172 PMC4175350

[8] Leung LL, Myles T, Morser J. Thrombin cleavage of osteopontin and the host Anti-Tumor immune response. Cancers (Basel). 2023;15:13. 10.3390/cancers15133480 PMC10340489 37444590

[9] Mi Z, Oliver T, Guo H, Gao C, Kuo PC. Thrombin-cleaved COOH(-) terminal osteopontin peptide binds with Cyclophilin C to CD147 in murine breast cancer cells. Cancer Res. 2007;67(9):4088–97. 17483319 10.1158/0008-5472.CAN-06-4066

[10] Song Z, Chen W, Athavale D, Ge X, Desert R, Das S, Han H, Nieto N. Osteopontin takes center stage in chronic liver disease. Hepatology. 2021;73(4):1594–608. 32986864 10.1002/hep.31582 PMC8106357

[11] Kumari A, Kashyap D, Garg VK. Osteopontin in cancer. Adv Clin Chem. 2024;118:87–110. 38280808 10.1016/bs.acc.2023.11.002

[12] Lu C, Liu Z, Klement JD, Yang D, Merting AD, Poschel D, Albers T, Waller JL, Shi H, Liu K. WDR5-H3K4me3 epigenetic axis regulates OPN expression to compensate PD-L1 function to promote pancreatic cancer immune escape. J Immunother Cancer. 2021;9:7. 10.1136/jitc-2021-002624 PMC8323468 34326167

[13] Chen Q, Shou P, Zhang L, Xu C, Zheng C, Han Y, Li W, Huang Y, Zhang X, Shao C, Roberts AI, Rabson AB, Ren G, Zhang Y, Wang Y, Denhardt DT, Shi Y. An osteopontin-integrin interaction plays a critical role in directing adipogenesis and osteogenesis by mesenchymal stem cells. Stem Cells. 2014;32(2):327–37. 24123709 10.1002/stem.1567 PMC3961005

[14] Liu L, Zhang R, Deng J, Dai X, Zhu X, Fu Q, Zhang H, Tong Z, Zhao P, Fang W, Zheng Y, Bao X. Construction of TME and identification of crosstalk between malignant cells and macrophages by SPP1 in hepatocellular carcinoma. Cancer Immunol Immunotherapy: CII. 2022;71(1):121–36. 34028567 10.1007/s00262-021-02967-8 PMC10992184

[15] Huang Z, Li Y, Liu Q, Chen X, Lin W, Wu W, Chen Z, Chen X, Pan Y, Qiu S. SPP1-mediated M2 macrophage polarization shapes the tumor microenvironment and enhances prognosis and immunotherapy guidance in nasopharyngeal carcinoma. Int Immunopharmacol. 2025;147:113944. 39742726 10.1016/j.intimp.2024.113944

[16] Bill R, Wirapati P, Messemaker M, Roh W, Zitti B, Duval F, Kiss M, Park JC, Saal TM, Hoelzl J, Tarussio D, Benedetti F, Tissot S, Kandalaft L, Varrone M, Ciriello G, McKee TA, Monnier Y, Mermod M, Blaum EM, Gushterova I, Gonye ALK, Hacohen N, Getz G, Mempel TR, Klein AM, Weissleder R, Faquin WC, Sadow PM, Lin D, Pai SI, Sade-Feldman M, Pittet MJ. CXCL9:SPP1 macrophage Polarity identifies a network of cellular programs that control human cancers. Science. 2023;381(6657):515–24. 37535729 10.1126/science.ade2292 PMC10755760

[17] Su X, Liang C, Chen R, Duan S. Deciphering tumor microenvironment: CXCL9 and SPP1 as crucial determinants of tumor-associated macrophage Polarity and prognostic indicators. Mol Cancer. 2024;23(1):13. 38217023 10.1186/s12943-023-01931-7 PMC10790255

[18] Klement JD, Paschall AV, Redd PS, Ibrahim ML, Lu C, Yang D, Celis E, Abrams SI, Ozato K, Liu K. An osteopontin/CD44 immune checkpoint controls CD8 + T cell activation and tumor immune evasion. J Clin Invest. 2018;128(12):5549–60. 30395540 10.1172/JCI123360 PMC6264631

[19] Morse C, Tabib T, Sembrat J, Buschur KL, Bittar HT, Valenzi E, Jiang Y, Kass DJ, Gibson K, Chen W, Mora A, Benos PV, Rojas M, Lafyatis R. Proliferating SPP1/MERTK-expressing macrophages in idiopathic pulmonary fibrosis. Eur Respir J 2019, *54* (2). 10.1183/13993003.02441-2018 PMC8025672 31221805

[20] Icer MA, Gezmen-Karadag M. The multiple functions and mechanisms of osteopontin. Clin Biochem. 2018;59:17–24. 30003880 10.1016/j.clinbiochem.2018.07.003

[21] Takahashi K, Tanabe K, Ohnuki M, Narita M, Ichisaka T, Tomoda K, Yamanaka S. Induction of pluripotent stem cells from adult human fibroblasts by defined factors. Cell. 2007;131(5):861–72. 18035408 10.1016/j.cell.2007.11.019

[22] Wu J, Izpisua Belmonte JC. Dynamic pluripotent stem cell States and their applications. Cell Stem Cell. 2015;17(5):509–25. 26544113 10.1016/j.stem.2015.10.009

[23] Batlle E, Clevers H. Cancer stem cells revisited. Nat Med. 2017;23(10):1124–34. 28985214 10.1038/nm.4409

[24] Triana-Martinez F, Loza MI, Dominguez E. Beyond tumor suppression: senescence in cancer stemness and tumor dormancy. Cells 2020, *9* (2). 10.3390/cells9020346 PMC7072600 32028565

[25] Chaffer CL, Brueckmann I, Scheel C, Kaestli AJ, Wiggins PA, Rodrigues LO, Brooks M, Reinhardt F, Su Y, Polyak K, Arendt LM, Kuperwasser C, Bierie B, Weinberg RA. Normal and neoplastic nonstem cells can spontaneously convert to a stem-like state. Proc Natl Acad Sci U S A. 2011;108(19):7950–5. 21498687 10.1073/pnas.1102454108 PMC3093533

[26] Chen C, Zhao S, Karnad A, Freeman JW. The biology and role of CD44 in cancer progression: therapeutic implications. J Hematol Oncol. 2018;11(1):64. 29747682 10.1186/s13045-018-0605-5 PMC5946470

[27] Hou W, Kong L, Hou Z, Ji H. CD44 is a prognostic biomarker and correlated with immune infiltrates in gastric cancer. BMC Med Genomics. 2022;15(1):225. 36316684 10.1186/s12920-022-01383-w PMC9620622

[28] Campbell ID, Humphries MJ. Integrin structure, activation, and interactions. Cold Spring Harb Perspect Biol 2011, *3* (3). 10.1101/cshperspect.a004994 PMC3039929 21421922

[29] Slack RJ, Macdonald SJF, Roper JA, Jenkins RG, Hatley RJD. Emerging therapeutic opportunities for integrin inhibitors. Nat Rev Drug Discov. 2022;21(1):60–78. 34535788 10.1038/s41573-021-00284-4 PMC8446727

[30] Jiang R, Lo J, Prell C, Lonnerdal B. Milk osteopontin promotes intestinal development by up-regulating the expression of integrin alphavbeta3 and CD44. FASEB J 2023, 37 (6), e22988. 10.1096/fj.202300092R 37219531

[31] Qian J, LeSavage BL, Hubka KM, Ma C, Natarajan S, Eggold JT, Xiao Y, Fuh KC, Krishnan V, Enejder A, Heilshorn SC, Dorigo O, Rankin EB. Cancer-associated mesothelial cells promote ovarian cancer chemoresistance through paracrine osteopontin signaling. J Clin Invest. 2021;131:16. 10.1172/JCI146186 PMC8363279 34396988

[32] Pang X, He X, Qiu Z, Zhang H, Xie R, Liu Z, Gu Y, Zhao N, Xiang Q, Cui Y. Targeting integrin pathways: mechanisms and advances in therapy. Signal Transduct Target Ther. 2023;8(1):1. 36588107 10.1038/s41392-022-01259-6 PMC9805914

[33] <2015 osteopontin promotes a cancer stem cell-like phenotype in hepatocellular carcinoma cells via an integrin–NF-κB–HIF-1α pathway.pdf>. 10.18632/oncotarget.3113 PMC4466639 25749383

[34] Pietras A, Katz AM, Ekstrom EJ, Wee B, Halliday JJ, Pitter KL, Werbeck JL, Amankulor NM, Huse JT, Holland EC. Osteopontin-CD44 signaling in the glioma perivascular niche enhances cancer stem cell phenotypes and promotes aggressive tumor growth. Cell Stem Cell. 2014;14(3):357–69. 24607407 10.1016/j.stem.2014.01.005 PMC3999042

[35] Okamoto H. Osteopontin and cardiovascular system. Mol Cell Biochem. 2007;300(1–2):1–7. 17136480 10.1007/s11010-006-9368-3

[36] Zeng B, Zhou M, Wu H, Xiong Z. SPP1 promotes ovarian cancer progression via integrin β1/FAK/AKT signaling pathway. OncoTargets Therapy. 2018;11:1333–43. 29559792 10.2147/OTT.S154215 PMC5856063

[37] Mondal S, Adhikari N, Banerjee S, Amin SA, Jha T. Matrix metalloproteinase-9 (MMP-9) and its inhibitors in cancer: A minireview. Eur J Med Chem. 2020;194:112260. 32224379 10.1016/j.ejmech.2020.112260

[38] Yang CL, Song R, Hu JW, Huang JT, Li NN, Ni HH, Li YK, Zhang J, Lu Z, Zhou M, Wang JD, Li MJ, Zhan GH, Peng T, Yu HP, Qi LN, Wang QY, Xiang BD. Integrating single-cell and bulk RNA sequencing reveals CK19 + cancer stem cells and their specific SPP1 + tumor-associated macrophage niche in HBV-related hepatocellular carcinoma. Hepatol Int. 2024;18(1):73–90. 38159218 10.1007/s12072-023-10615-9

[39] Meng X, Cui J, He G. Bcl-2 Is Involved in Cardiac Hypertrophy through PI3K-Akt Pathway. *Biomed Res Int* 2021, *2021*, 6615502. 10.1155/2021/6615502 PMC7979306 33778070

[40] Zhang Y, Li S, Cui X, Wang Y. microRNA-944 inhibits breast cancer cell proliferation and promotes cell apoptosis by reducing SPP1 through inactivating the PI3K/Akt pathway. Apoptosis. 2023;28(11–12):1546–63. 37486406 10.1007/s10495-023-01870-0

[41] Matsubara E, Yano H, Pan C, Komohara Y, Fujiwara Y, Zhao S, Shinchi Y, Kurotaki D, Suzuki M. The significance of SPP1 in lung cancers and its impact as a marker for protumor Tumor-Associated macrophages. Cancers (Basel). 2023;15:8. 10.3390/cancers15082250 PMC10136569 37190178

[42] Lou H, Wu LQ, Wang H, Wei RL, Cheng JW. The potential role of osteopontin in the pathogenesis of graves’ ophthalmopathy. Invest Ophthalmol Vis Sci. 2021;62(12):18. 34546326 10.1167/iovs.62.12.18 PMC8458783

[43] Wang L, Zhang L, Zhao L, Shao S, Ning Q, Jing X, Zhang Y, Zhao F, Liu X, Gu S, Zhao X, Luo M. VEGFA/NRP-1/GAPVD1 axis promotes progression and cancer stemness of triple-negative breast cancer by enhancing tumor cell-macrophage crosstalk. Int J Biol Sci. 2024;20(2):446–63. 38169627 10.7150/ijbs.86085 PMC10758102

[44] Mercurio AM. VEGF/Neuropilin signaling in cancer stem cells. Int J Mol Sci 2019, *20* (3). 10.3390/ijms20030490 PMC6387347 30678134

[45] Liu X, Qin J, Nie J, Gao R, Hu S, Sun H, Wang S, Pan Y. ANGPTL2 + cancer-associated fibroblasts and SPP1 + macrophages are metastasis accelerators of colorectal cancer. Front Immunol. 2023;14:1185208. 37691929 10.3389/fimmu.2023.1185208 PMC10483401

[46] Yi X, Luo L, Zhu Y, Deng H, Liao H, Shen Y, Zheng Y. SPP1 facilitates cell migration and invasion by targeting COL11A1 in lung adenocarcinoma. Cancer Cell Int. 2022;22(1):324. 36266702 10.1186/s12935-022-02749-x PMC9583566

[47] Yunna C, Mengru H, Lei W, Weidong C. Macrophage M1/M2 polarization. Eur J Pharmacol. 2020;877:173090. 32234529 10.1016/j.ejphar.2020.173090

[48] Zhang Y, Du W, Chen Z, Xiang C. Upregulation of PD-L1 by SPP1 mediates macrophage polarization and facilitates immune escape in lung adenocarcinoma. Exp Cell Res. 2017;359(2):449–57. 28830685 10.1016/j.yexcr.2017.08.028

[49] Yu S, Chen M, Xu L, Mao E, Sun S. A senescence-based prognostic gene signature for colorectal cancer and identification of the role of SPP1-positive macrophages in tumor senescence. Front Immunol. 2023;14:1175490. 37090726 10.3389/fimmu.2023.1175490 PMC10115976

[50] Wu J, Shen Y, Zeng G, Liang Y, Liao G. SPP1(+) TAM subpopulations in tumor microenvironment promote intravasation and metastasis of head and neck squamous cell carcinoma. Cancer Gene Ther. 2024;31(2):311–21. 38052857 10.1038/s41417-023-00704-0

[51] Moldogazieva NT, Mokhosoev IM, Zavadskiy SP, Terentiev AA. Proteomic profiling and artificial intelligence for hepatocellular carcinoma translational medicine. Biomedicines 2021, *9* (2). 10.3390/biomedicines9020159 PMC7914649 33562077

[52] Wang C, Li Y, Wang L, Han Y, Gao X, Li T, Liu M, Dai L, Du R. SPP1 represents a therapeutic target that promotes the progression of oesophageal squamous cell carcinoma by driving M2 macrophage infiltration. Br J Cancer. 2024;130(11):1770–82. 38600327 10.1038/s41416-024-02683-x PMC11130281

[53] Deng G, Zeng F, Su J, Zhao S, Hu R, Zhu W, Hu S, Chen X, Yin M. BET inhibitor suppresses melanoma progression via the noncanonical NF-κB/SPP1 pathway. Theranostics. 2020;10(25):11428–43. 33052224 10.7150/thno.47432 PMC7546000

[54] Liu Y, Xun Z, Ma K, Liang S, Li X, Zhou S, Sun L, Liu Y, Du Y, Guo X, Cui T, Zhou H, Wang J, Yin D, Song R, Zhang S, Cai W, Meng F, Guo H, Zhang B, Yang D, Bao R, Hu Q, Wang J, Ye Y, Liu L. Identification of a tumour immune barrier in the HCC microenvironment that determines the efficacy of immunotherapy. J Hepatol. 2023;78(4):770–82. 36708811 10.1016/j.jhep.2023.01.011

[55] Yue B, Xiong D, Chen J, Yang X, Zhao J, Shao J, Wei D, Gao F, Huang M, Chen J. SPP1 induces idiopathic pulmonary fibrosis and NSCLC progression via the PI3K/Akt/mTOR pathway. Respir Res. 2024;25(1):362. 39369217 10.1186/s12931-024-02989-7 PMC11456247

[56] Chen Z, Chen M, Yang C, Wang J, Gao Y, Feng Y, Yuan D, Chen P. Integration of Multi-Scale profiling and machine learning reveals the prognostic role of extracellular Matrix-Related Cancer-Associated fibroblasts in lung adenocarcinoma. Int J Med Sci. 2025;22(12):2956–72. 40657391 10.7150/ijms.113580 PMC12243870

[57] Chen C, Guo Q, Liu Y, Hou Q, Liao M, Guo Y, Zang Y, Wang F, Liu H, Luan X, Liang Y, Guan Z, Li Y, Liu H, Dong X, Zhang X, Liu J, Xu Q. Single-cell and Spatial transcriptomics reveal POSTN(+) cancer-associated fibroblasts correlated with immune suppression and tumour progression in non-small cell lung cancer. Clin Transl Med 2023, 13 (12), e1515. 10.1002/ctm2.1515 PMC10731139 38115703

[58] Hong YK, Hwang DY, Yang CC, Cheng SM, Chen PC, Aala WJ, H ICH, Evans ST, Onoufriadis A, Liu SL, Lin YC, Chang YH, Lo TK, Hung KS, Lee YC, Tang MJ, Lu KQ, McGrath JA, Hsu CK. Profibrotic subsets of SPP1(+) macrophages and POSTN(+) fibroblasts contribute to fibrotic scarring in acne keloidalis. J Invest Dermatol. 2024;144(7):1491–e150410. 38218364 10.1016/j.jid.2023.12.014

[59] Wang H, Liang Y, Liu Z, Zhang R, Chao J, Wang M, Liu M, Qiao L, Xuan Z, Zhao H, Lu L. POSTN(+) cancer-associated fibroblasts determine the efficacy of immunotherapy in hepatocellular carcinoma. J Immunother Cancer. 2024;12:7. 10.1136/jitc-2023-008721 PMC11284881 39067872

[60] Su Z, He Y, You L, Chen J, Zhang G, Liu Z. SPP1 + macrophages and FAP + fibroblasts promote the progression of pMMR gastric cancer. Sci Rep. 2024;14(1):26221. 39482333 10.1038/s41598-024-76298-w PMC11528032

[61] He H, Chen S, Fan Z, Dong Y, Wang Y, Li S, Sun X, Song Y, Yang J, Cao Q, Jiang J, Wang X, Wen W, Wang H. Multi-dimensional single-cell characterization revealed suppressive immune microenvironment in AFP-positive hepatocellular carcinoma. Cell Discov. 2023;9(1):60. 37336873 10.1038/s41421-023-00563-x PMC10279759

[62] Brina D, Ponzoni A, Troiani M, Cali B, Pasquini E, Attanasio G, Mosole S, Mirenda M, D’Ambrosio M, Colucci M, Guccini I, Revandkar A, Alajati A, Tebaldi T, Donzel D, Lauria F, Parhizgari N, Valdata A, Maddalena M, Calcinotto A, Bolis M, Rinaldi A, Barry S, Ruschoff JH, Sabbadin M, Sumanasuriya S, Crespo M, Sharp A, Yuan W, Grinu M, Boyle A, Miller C, Trotman L, Delaleu N, Fassan M, Moch H, Viero G, de Bono J, Alimonti A. The Akt/mTOR and MNK/eIF4E pathways rewire the prostate cancer translatome to secrete HGF, SPP1 and BGN and recruit suppressive myeloid cells. Nat Cancer. 2023;4(8):1102–21. 37460872 10.1038/s43018-023-00594-z PMC11331482

[63] Xu X, Lin J, Wang J, Wang Y, Zhu Y, Guo J. SPP1 expression indicates outcome of immunotherapy plus tyrosine kinase Inhibition in advanced renal cell carcinoma. Hum Vaccin Immunother. 2024;20(1):2350101. 38738709 10.1080/21645515.2024.2350101 PMC11093034

[64] Denhardt DT, Mistretta D, Chambers AF, Krishna S, Porter JF, Raghuram S, Rittling SR. Transcriptional regulation of osteopontin and the metastatic phenotype: evidence for a Ras-activated enhancer in the human OPN promoter. Clin Exp Metastasis. 2003;20(1):77–84. 12650610 10.1023/a:1022550721404

[65] Liu SJ, Hu GF, Liu YJ, Liu SG, Gao H, Zhang CS, Wei YY, Xue Y, Lao WD. Effect of human osteopontin on proliferation, transmigration and expression of MMP-2 and MMP-9 in osteosarcoma cells. Chin Med J. 2004;117(2):235–40. 14975209

[66] Berge G, Pettersen S, Grotterød I, Bettum IJ, Boye K, Mælandsmo GM. Osteopontin–an important downstream effector of S100A4-mediated invasion and metastasis. Int J Cancer. 2011;129(4):780–90. 20957651 10.1002/ijc.25735

[67] Dalla-Torre CA, Yoshimoto M, Lee CH, Joshua AM, de Toledo SR, Petrilli AS, Andrade JA, Chilton-MacNeill S, Zielenska M, Squire JA. Effects of THBS3, SPARC and SPP1 expression on biological behavior and survival in patients with osteosarcoma. BMC Cancer. 2006;6:237. 17022822 10.1186/1471-2407-6-237 PMC1609181

[68] Colia V, Stacchiotti S. Medical treatment of advanced Chordomas. Eur J Cancer. 2017;83:220–8. 28750274 10.1016/j.ejca.2017.06.038

[69] Pan HW, Ou YH, Peng SY, Liu SH, Lai PL, Lee PH, Sheu JC, Chen CL, Hsu HC. Overexpression of osteopontin is associated with intrahepatic metastasis, early recurrence, and poorer prognosis of surgically resected hepatocellular carcinoma. Cancer. 2003;98(1):119–27. 12833464 10.1002/cncr.11487

[70] Kim J, Ki SS, Lee SD, Han CJ, Kim YC, Park SH, Cho SY, Hong YJ, Park HY, Lee M, Jung HH, Lee KH, Jeong SH. Elevated plasma osteopontin levels in patients with hepatocellular carcinoma. Am J Gastroenterol. 2006;101(9):2051–9. 16848813 10.1111/j.1572-0241.2006.00679.x

[71] El-Tanani MK. Role of osteopontin in cellular signaling and metastatic phenotype. Front Bioscience: J Virtual Libr. 2008;13:4276–84. 10.2741/3004 18508510

[72] Wang J, Yang K, Yang X, Jin T, Tian Y, Dai C, Xu F. HHLA2 promotes hepatoma cell proliferation, migration, and invasion via SPP1/PI3K/AKT signaling pathway. Mol Carcinog. 2024;63(7):1275–87. 38578157 10.1002/mc.23723

[73] Liu Y, Zhang L, Ju X, Wang S, Qie J. Single-Cell transcriptomic analysis reveals Macrophage-Tumor crosstalk in hepatocellular carcinoma. Front Immunol. 2022;13:955390. 35958556 10.3389/fimmu.2022.955390 PMC9359093

[74] Amilca-Seba K, Tan TZ, Thiery JP, Louadj L, Thouroude S, Bouygues A, Sabbah M, Larsen AK, Denis JA. Osteopontin (OPN/SPP1), a mediator of tumor Progression, is regulated by the mesenchymal transcription factor Slug/SNAI2 in colorectal cancer (CRC). Cells. 2022;11:11. 10.3390/cells11111808 PMC9180003 35681502

[75] Xu C, Sun L, Jiang C, Zhou H, Gu L, Liu Y, Xu Q. SPP1, analyzed by bioinformatics methods, promotes the metastasis in colorectal cancer by activating EMT pathway. Biomed pharmacotherapy = Biomedecine Pharmacotherapie. 2017;91:1167–77. 10.1016/j.biopha.2017.05.056 28531945

[76] Qi J, Sun H, Zhang Y, Wang Z, Xun Z, Li Z, Ding X, Bao R, Hong L, Jia W, Fang F, Liu H, Chen L, Zhong J, Zou D, Liu L, Han L, Ginhoux F, Liu Y, Ye Y, Su B. Single-cell and Spatial analysis reveal interaction of FAP(+) fibroblasts and SPP1(+) macrophages in colorectal cancer. Nat Commun. 2022;13(1):1742. 35365629 10.1038/s41467-022-29366-6 PMC8976074

[77] Choe EK, Yi JW, Chai YJ, Park KJ. Upregulation of the adipokine genes ADIPOR1 and SPP1 is related to poor survival outcomes in colorectal cancer. J Surg Oncol. 2018;117(8):1833–40. 29761507 10.1002/jso.25078

[78] Xie Z, Zheng G, Niu L, Du K, Li R, Dan H, Duan L, Wu H, Ren G, Dou X, Dai S, Feng F, Zhang J, Zheng J. SPP1 (+) macrophages in colorectal cancer: markers of malignancy and promising therapeutic targets. Genes Dis. 2025;12(3):101340. 40092488 10.1016/j.gendis.2024.101340 PMC11907465

[79] Zhao K, Ma Z, Zhang W. Comprehensive analysis to identify SPP1 as a prognostic biomarker in cervical cancer. Front Genet. 2021;12:732822. 35058964 10.3389/fgene.2021.732822 PMC8764398

[80] Qin S, Yi L, Liang Y, Chen Y, Wang W, Liao Y, Zhang C, Huang H, Huang J, Yao S. Biological and clinicopathological characteristics of OPN in cervical cancers. Front Genet. 2022;13:836509. 35669197 10.3389/fgene.2022.836509 PMC9163571

[81] Chen X, Xiong D, Ye L, Yang H, Mei S, Wu J, Chen S, Mi R. SPP1 Inhibition improves the cisplatin chemo-sensitivity of cervical cancer cell lines. Cancer Chemother Pharmacol. 2019;83(4):603–13. 30627777 10.1007/s00280-018-3759-5

[82] Poleboyina PK, Alagumuthu M, Pasha A, Ravinder D, Pasumarthi D, Pawar SC. Entrectinib a plausible inhibitor for osteopontin (SPP1) in cervical Cancer-Integrated bioinformatic approach. Appl Biochem Biotechnol. 2023;195(12):7766–95. 37086377 10.1007/s12010-023-04541-7

[83] Li B, Li X, Yang Q, Jiang Y, Zhang Q, Zhang J, Cui W, Xu F. Overexpression of SPP1 is a prognostic indicator of immune infiltration in lung adenocarcinoma. Aging. 2024;16(3):2953–77. 38329443 10.18632/aging.205526 PMC10911343

[84] Tang H, Chen J, Han X, Feng Y, Wang F. Upregulation of SPP1 is a marker for poor lung cancer prognosis and contributes to cancer progression and cisplatin resistance. Front Cell Dev Biol. 2021;9:646390. 33996808 10.3389/fcell.2021.646390 PMC8116663

[85] Wang X, Zhang F, Yang X, Xue M, Li X, Gao Y, Liu L. Secreted phosphoprotein 1 (SPP1) contributes to Second-Generation EGFR tyrosine kinase inhibitor resistance in Non-Small cell lung cancer. Oncol Res. 2019;27(8):871–7. 30832751 10.3727/096504018X15426271404407 PMC7848392

[86] Dong B, Wu C, Huang L, Qi Y. Macrophage-Related SPP1 as a potential biomarker for early lymph node metastasis in lung adenocarcinoma. Front Cell Dev Biology. 2021;9:739358. 10.3389/fcell.2021.739358 PMC8502925 34646827

[87] Xiao Z, Nian Z, Zhang M, Liu Z, Zhang P, Zhang Z. Single-cell and bulk RNA-sequencing reveal SPP1 and CXCL12 as cell-to-cell communication markers to predict prognosis in lung adenocarcinoma. *Environmental toxicology* 2024. 10.1002/tox.24297 38622884

[88] Wang Z, Leverenz K, Thomas DG, Myers AL, Chang AC, Orringer MB, Giordano TJ, Lin J, Beer DG, Lin L. Abstract 3879: osteopontin (SPP1/OPN) alternative splice variants and metastatic potential in esophageal adenocarcinoma. Cancer Res. 2013;73(8Supplement):3879–3879.

[89] Song Y, Wang X, Wang F, Peng X, Li P, Liu S, Zhang D. Identification of four genes and biological characteristics of esophageal squamous cell carcinoma by integrated bioinformatics analysis. Cancer Cell Int. 2021;21(1):123. 33602210 10.1186/s12935-021-01814-1 PMC7890804

[90] Wang M, Sun X, Xin H, Wen Z, Cheng Y. SPP1 promotes radiation resistance through JAK2/STAT3 pathway in esophageal carcinoma. Cancer Med. 2022;11(23):4526–43. 35593388 10.1002/cam4.4840 PMC9741975

[91] Gao W, Liu D, Sun H, Shao Z, Shi P, Li T, Yin S, Zhu T. SPP1 is a prognostic related biomarker and correlated with tumor-infiltrating immune cells in ovarian cancer. BMC Cancer. 2022;22(1):1367. 36585688 10.1186/s12885-022-10485-8 PMC9805166

[92] Chandran UR, Ma C, Dhir R, Bisceglia M, Lyons-Weiler M, Liang W, Michalopoulos G, Becich M, Monzon FA. Gene expression profiles of prostate cancer reveal involvement of multiple molecular pathways in the metastatic process. BMC Cancer. 2007;7:64. 17430594 10.1186/1471-2407-7-64 PMC1865555

[93] Messex JK, Byrd CJ, Thomas MU, Liou GY. Macrophages cytokine Spp1 increases growth of prostate intraepithelial neoplasia to promote prostate tumor progression. Int J Mol Sci. 2022;23:8. 10.3390/ijms23084247 PMC9027984 35457063

[94] Pang X, Zhang J, He X, Gu Y, Qian BZ, Xie R, Yu W, Zhang X, Li T, Shi X, Zhou Y, Cui Y. SPP1 Promotes Enzalutamide Resistance and Epithelial-Mesenchymal-Transition Activation in Castration-Resistant Prostate Cancer via PI3K/AKT and ERK1/2 Pathways. *Oxidative medicine and cellular longevity* 2021, *2021*, 5806602. 10.1155/2021/5806602 PMC8556132 34721759

[95] Ouyang L, Sun MM, Zhou PS, Ren YW, Liu XY, Wei WY, Song ZS, Lu K, Yang LX. LncRNA FOXD1-AS1 regulates pancreatic cancer stem cell properties and 5-FU resistance by regulating the miR-570-3p/SPP1 axis as a CeRNA. Cancer Cell Int. 2024;24(1):4. 38167126 10.1186/s12935-023-03181-5 PMC10763109

[96] Yang Y, Gong Y, Ding Y, Sun S, Bai R, Zhuo S, Zhang Z. LINC01133 promotes pancreatic ductal adenocarcinoma epithelial-mesenchymal transition mediated by SPP1 through binding to Arp3. Cell Death Dis. 2024;15(7):492. 38987572 10.1038/s41419-024-06876-3 PMC11237081

[97] Nallasamy P, Nimmakayala RK, Karmakar S, Leon F, Seshacharyulu P, Lakshmanan I, Rachagani S, Mallya K, Zhang C, Ly QP, Myers MS, Josh L, Grabow CE, Gautam SK, Kumar S, Lele SM, Jain M, Batra SK, Ponnusamy MP. Pancreatic tumor microenvironment factor promotes cancer stemness via SPP1-CD44 axis. Gastroenterology. 2021;161(6):1998–2013. e7. 34418441 10.1053/j.gastro.2021.08.023 PMC10069715

[98] Rabjerg M, Guerra B, Oliván-Viguera A, Mikkelsen MLN, Köhler R, Issinger O-G, Marcussen N. Nuclear localization of the CK2α-subunit correlates with poor prognosis in clear cell renal cell carcinoma. Oncotarget. 2016;8(1):1613–27. 10.18632/oncotarget.13693 PMC5352082 27906674

[99] Wang Y, Su J, Wang Y, Fu D, Ideozu JE, Geng H, Cui Q, Wang C, Chen R, Yu Y, Niu Y, Yue D. The interaction of YBX1 with G3BP1 promotes renal cell carcinoma cell metastasis via YBX1/G3BP1-SPP1- NF-kappaB signaling axis. J Exp Clin Cancer Res. 2019;38(1):386. 31481087 10.1186/s13046-019-1347-0 PMC6720408

[100] Zhang J, Liu F, Guo W, Bi X, Yuan S, Shayiti F, Pan T, Li K, Chen P. Single-cell transcriptome sequencing reveals aberrantly activated inter-tumor cell signaling pathways in the development of clear cell renal cell carcinoma. J Translational Med 2024, *22* (1). 10.1186/s12967-023-04818-9 PMC10775677 38191424

[101] Assidi M, Gomaa W, Jafri M, Hanbazazh M, Al-Ahwal M, Pushparaj P, Al-Harbi A, Al-Qahtani M, Buhmeida A, Al-Maghrabi J. Prognostic value of osteopontin (SPP1) in colorectal carcinoma requires a personalized molecular approach. Tumour Biology: J Int Soc Oncodevelopmental Biology Med. 2019;41(9):1010428319863627. 10.1177/1010428319863627 31500540

[102] Al Maghrabi H, Gomaa W, Al-Maghrabi J. Increased osteopontin expression in endometrial carcinoma is associated with better survival outcome. Ginekologia Polska. 2020;91(2):73–8. 32141052 10.5603/GP.2020.0020

[103] Collins AL, Rock J, Malhotra L, Frankel WL, Bloomston M. Osteopontin expression is associated with improved survival in patients with pancreatic adenocarcinoma. Ann Surg Oncol. 2012;19(8):2673–8. 22461132 10.1245/s10434-012-2337-z PMC3407314

[104] Yu A, Guo K, Qin Q, Xing C, Zu X. Clinicopathological and prognostic significance of osteopontin expression in patients with prostate cancer: a systematic review and meta-analysis. Biosci Rep. 2021;41:8. 10.1042/BSR20203531 PMC8350436 33635319

[105] Shi SM, Su ZB, Zhao JJ, Yu DJ, Tu JW, Zhu JQ, Zhao JP, Sheng L, Wang SB, Sheng YJ, Chen HJ, Tian JH, Zhang Y, Wang J. Increased osteopontin protein expression May be correlated with poor prognosis in non-small-cell lung cancer: A meta analysis. J Cancer Res Ther. 2016;12(1):277–82. 27072251 10.4103/0973-1482.150362

[106] Zhang X, Tsukamoto T, Mizoshita T, Ban H, Suzuki H, Toyoda T, Tatematsu M. Expression of osteopontin and CDX2: indications of phenotypes and prognosis in advanced gastric cancer. Oncol Rep. 2009;21(3):609–13. 19212618

[107] Göthlin Eremo A, Lagergren K, Othman L, Montgomery S, Andersson G, Tina E. Evaluation of SPP1/osteopontin expression as predictor of recurrence in Tamoxifen treated breast cancer. Sci Rep. 2020;10(1):1451. 31996744 10.1038/s41598-020-58323-w PMC6989629

[108] Wong JPC, Wei R, Lyu P, Tong OLH, Zhang SD, Wen Q, Yuen HF, El-Tanani M, Kwok HF. Clinical and in vitro analysis of osteopontin as a prognostic indicator and unveil its potential downstream targets in bladder cancer. Int J Biol Sci. 2017;13(11):1373–86. 29209142 10.7150/ijbs.21457 PMC5715521

[109] Trehan R, Huang P, Zhu XB, Wang X, Soliman M, Strepay D, Nur A, Kedei N, Arhin M, Ghabra S, Rodriguez-Matos F, Benmebarek MR, Ma C, Korangy F, Greten TF. SPP1 + macrophages cause exhaustion of tumor-specific T cells in liver metastases. Nat Commun. 2025;16(1):4242. 40335453 10.1038/s41467-025-59529-0 PMC12059142

[110] Ebeling S, Kowalczyk A, Perez-Vazquez D, Mattiola I. Regulation of tumor angiogenesis by the crosstalk between innate immunity and endothelial cells. Front Oncol. 2023;13:1171794. 37234993 10.3389/fonc.2023.1171794 PMC10206118

[111] Muchlinska A, Nagel A, Popeda M, Szade J, Niemira M, Zielinski J, Skokowski J, Bednarz-Knoll N, Zaczek AJ. Alpha-smooth muscle actin-positive cancer-associated fibroblasts secreting osteopontin promote growth of luminal breast cancer. Cell Mol Biol Lett. 2022;27(1):45. 35690734 10.1186/s11658-022-00351-7 PMC9188043

[112] Matusiak M, van Hickey JW, Lu IDGP, Kidzinski G, Zhu L, Colburg S, Luca DRC, Phillips B, Brubaker DJ, Charville SW, Shen GW, Loh J, Okwan-Duodu KM, Nolan DK, Newman GP, West AM, van de Rijn RB. Spatially segregated macrophage populations predict distinct outcomes in colon cancer. Cancer Discov. 2024;14(8):1418–39. 38552005 10.1158/2159-8290.CD-23-1300 PMC11294822

[113] Deng G, Zeng F, Su J, Zhao S, Hu R, Zhu W, Hu S, Chen X, Yin M. BET inhibitor suppresses melanoma progression via the noncanonical NF-kappaB/SPP1 pathway. Theranostics. 2020;10(25):11428–43. 33052224 10.7150/thno.47432 PMC7546000

[114] <1998 Altered wound healing in mice lacking a functional osteopontin gene (spp1).pdf&gt. 10.1172/JCI1122 PMC508725 9525990

[115] <. Anti-CD44 antibody treatment lowers hyperglycemia and improves insulin Resistance, adipose Inflammation, and hepatic steatosis in Diet-. Induced Obese Mice.pdf>; 2015. 10.2337/db14-0149 PMC4392898 25294945

[116] Yadav MC, Huesa C, Narisawa S, Hoylaerts MF, Moreau A, Farquharson C, Millan JL. Ablation of osteopontin improves the skeletal phenotype of phospho1(-/-) mice. J Bone Min Res. 2014;29(11):2369–81. 10.1002/jbmr.2281 PMC5247257 24825455

[117] Doroshow DB, Bhalla S, Beasley MB, Sholl LM, Kerr KM, Gnjatic S, Wistuba I, Rimm DL, Tsao MS, Hirsch FR. PD-L1 as a biomarker of response to immune-checkpoint inhibitors. Nat Rev Clin Oncol. 2021;18(6):345–62. 33580222 10.1038/s41571-021-00473-5

[118] Chen K, Li Y, Ni J, Yang X, Zhou Y, Pang Y, Ye R, Chen H, Yu S, Wang P, Zhu Z. Identification of a novel subtype of SPP1 + macrophages expressing sirpalpha: implications for tumor immune evasion and treatment response prediction. Exp Hematol Oncol. 2024;13(1):119. 39696410 10.1186/s40164-024-00587-3 PMC11657677

[119] Darnell JE Jr., Kerr IM, Stark GR. Jak-STAT pathways and transcriptional activation in response to IFNs and other extracellular signaling proteins. Science. 1994;264(5164):1415–21. 8197455 10.1126/science.8197455

[120] Antoniu SA, Kolb MR. Intedanib, a triple kinase inhibitor of VEGFR, FGFR and PDGFR for the treatment of cancer and idiopathic pulmonary fibrosis. IDrugs. 2010;13(5):332–45. 20432191

[121] Capdevila J, Carrato A, Tabernero J, Grande E. What could nintedanib (BIBF 1120), a triple inhibitor of VEGFR, PDGFR, and FGFR, add to the current treatment options for patients with metastatic colorectal cancer? Crit Rev Oncol Hematol. 2014;92(2):83–106. 24924525 10.1016/j.critrevonc.2014.05.004

[122] Syed YY. Surufatinib: first approval. Drugs. 2021;81(6):727–32. 33788183 10.1007/s40265-021-01489-y

